# Internalization of Lipid-Coated Gold Nanocomposites and Gold Nanoparticles by Mouse SC-1 Fibroblasts in Monolayer and Spheroids

**DOI:** 10.3390/nano15181419

**Published:** 2025-09-15

**Authors:** Julia E. Poletaeva, Boris P. Chelobanov, Anna V. Epanchintseva, Anastasiya V. Tupitsyna, Ilya S. Dovydenko, Elena I. Ryabchikova

**Affiliations:** 1 Institute of Chemical Biology and Fundamental Medicine, Siberian Branch of Russian Academy of Science, Lavrent’ev avenue. 8, 630090 Novosibirsk, Russia; poletaeva@niboch.nsc.ru (J.E.P.); chelobanov.bp@mipt.ru (B.P.C.); annaepanch@niboch.nsc.ru (A.V.E.); tupitsyna@niboch.nsc.ru (A.V.T.); dovydenko_il@niboch.nsc.ru (I.S.D.); 2 Moscow Center for Advanced Studies, Kulakova Street 20, 123592 Moscow, Russia

**Keywords:** SC-1 fibroblasts, monolayer and spheroid, lipid-enveloped nanocomposite MLNC, AuNPs, ultrastructure, clathrin-mediated endocytosis, macropinocytosis, penetration depth into spheroid

## Abstract

In this study, we have established that unique composite particles (MLNCs) carried siRNA on a gold core and were covered with a lipid shell. MLNCs successfully delivered siRNa into cells in the presence of serum. We developed the photofixation method, allowing us to obtain MLNCs bearing a fixed protein corona. To understand the mechanisms of the influence that the protein corona has on the interaction of particles with cells, it is necessary to study the interaction of “naked” MLNCs with cells. This study aimed to examine the pathways of MLNC penetration into SC-1 fibroblasts used to confirm the efficacy of siRNA delivery. We studied fibroblasts in monolayer and spheroid form, and citrate AuNPs were used as a comparison particle. The same particles served as cores for MLNCs. The obtained results showed active penetration by clathrin-mediated endocytosis of “naked” MLNCs into SC-1 fibroblasts, regardless of the form of cultivation. AuNPs penetrated into monolayer fibroblasts by macropinocytosis and into spheroids by clathrin-mediated endocytosis. The penetration depth into the spheroids was about 40 μm for both types of particles (spheroid size was 350–400 μm). The particles migrated through the intercellular spaces, passing through intercellular contacts.

## 1. Introduction

Gold nanoparticles are a recognized agent of nanomedicine, which are used both as individual AuNPs and as part of various nanocomposites. The unique physicochemical properties of AuNPs, which ensure their use in various areas of nanomedicine, are described in numerous publications and reviews [1,2,3,4]. Based on AuNPs, we have developed composites carrying oligonucleotides non-covalently bound to the surface of the AuNPs, including therapeutic ones [5,6,7]. The multilayer nanocomposite (MLNC) we designed demonstrated the ability to effectively deliver siRNA into cells [5,6].

Using MLNCs, we obtained for the first time a fixed full protein corona using our own photofixation method and determined the protein composition of the soft corona [8], which plays a key role in interaction with cells [9,10,11]. “Control” of the composition of the soft protein corona is one of the most important tasks in nanomedicine, and one which has not yet been solved, largely due to the lack of methods for studying its composition [12,13,14]. Currently, two methods of fixing the protein corona and subsequent determination of its composition have been published: the click chemistry method [15], and the photofixation method (our study) [8].

The task of determining the influence of the protein corona on interaction with cells obviously follows from the global task of learning to “control” the composition of the protein corona, which is necessary for targeted drug delivery. Our nanocomposite MLNC, on which the protein corona was obtained and studied, looks like a good candidate for studying the interaction of “crowned” nanoparticles with cells: its physicochemical properties have been established, and it carries siRNA on the surface of the gold core, the effectiveness of which has been demonstrated in vitro. The presence of AuNPs inside the composite allows visualization of MLNCs on the surface and inside cells using the TEM method. However, there is a major gap in this “true masterpiece”: we do not know how MLNCs penetrate into target cells.

To fill this gap, this study was conducted to examine the pathways of MLNC penetration into cells, which in the future will allow us to evaluate the effect of the protein corona on the efficiency of siRNA delivery. These cells are fibroblasts of the SC-1 line carrying the gene of the green fluorescent protein (GFP) [16], which is the target of siRNA carried by MLNCs particles. In this study we used two variants of SC-1 fibroblasts: monolayer and spheroids.

Obviously, we applied transmission electron microscopy (TEM) to visualize penetration of the MLNCs and AuNPs, which served as “comparison particle”, into SC-1 fibroblasts. TEM is an indispensable method for studying the interaction of nanoparticles with cells, providing direct visualization of both cell structures and the NPs [17,18,19,20,21]. Despite the emergence of new methods for studying endocytosis, TEM remains an essential tool in the study of endocytic processes and facilitates the unambiguous identification of cellular structures involved in endocytosis [19,21].

The obtained results showed that MLNC penetration into fibroblasts occurred mainly via clathrin-mediated endocytosis. The routes of AuNP internalization depended on the form of fibroblast culture: macropinocytosis was more often recorded in the monolayer, while clathrin-dependent endocytosis was observed in spheroids.

## 2. Materials and Methods

### 2.1. Physicochemical Characterization of Nanoparticles

In this work, we used two types of NPs: AuNPs obtained by the citrate reduction method [22], and MLNCs, the assembly of which was fully described in our previous publication [7]. We used only freshly prepared suspensions of MLNCs in this work.

The hydrodynamic diameters of the NPs were estimated using a Malvern Zetasizer Nano-ZS (Malvern Instruments, Malvern, UK). The parameters of each sample were determined at least five times. The hydrodynamic diameters of the AuNPs and MLNCs in an aqueous suspension were 17.4 ± 0.4 nm and 210.4 ± 86.79 nm, respectively. The values of zeta potential (net charge) were −33.6 ± 2.0 mV and −35.3 ± 5.6 mV, respectively.

To control the quality of the suspensions and determine the sizes of the NPs, the samples of AuNPs or MLNCs were adsorbed onto copper grids coated with a formvar film for one minute, then a grid was dried with filter paper. The MLNC samples were contrasted with 0.5% uranyl acetate (EMS, Hatfield, PA, USA). The samples were then examined using a JEM 1400 TEM (JEOL, Tokyo, Japan), which was equipped with a Veleta digital camera (EM SIS, Münster, Germany). The NP sizes were measured using the iTEM version 5.2 software program (EM SIS, Münster, Germany).

**Figure 1:**
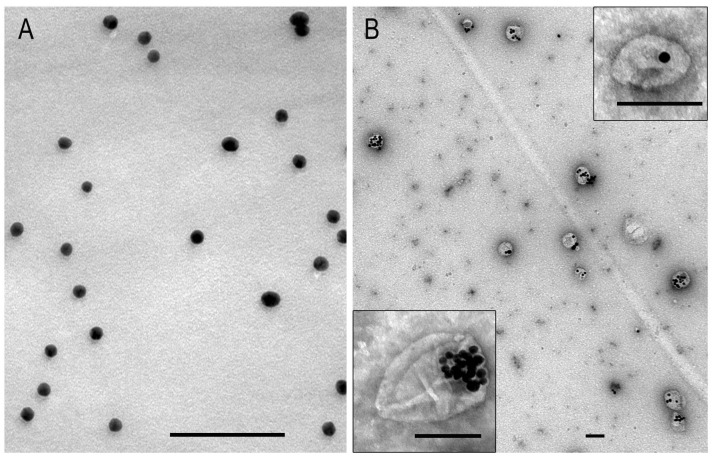
Representative images of AuNPs (**A**) and MLNCs (**B**) in TEM. MLNC sample was negatively stained with 0.5% uranyl acetate. The scale bars correspond to 100 nm.

Analysis of the AuNP suspension by TEM (Figure 1A) revealed uniform spherical particles with a high electron density (d = 13.4 ± 1.3 nm). In contrast, the MLNC suspension contained polymorphic particles with a distinct envelope of low electron density containing one to ten electron-dense cores, predominantly arranged asymmetrically (see Figure 1B). The prepared AuNPs and MLNCs were the same size and properties as those used in previous studies [7,23].

### 2.2. Cell Lines and Spheroid Preparation

The mouse embryonic fibroblast line (SC-1 R780, hereinafter referred to as SC-1), which carries a stably integrated GFP gene in its genome [16], was kindly provided by Dr. V. S. Prasolov (Engelhardt Institute of Molecular Biology, RAS, Moscow). The monolayers of the fibroblasts were cultured in DMEM medium, supplemented with 10% FBS (Thermo Fisher Scientific, Waltham, MA, USA), 100 U/mL penicillin, and 100 U/mL streptomycin (Thermo Fisher Scientific, Waltham, MA, USA), in a 5% CO_2_ atmosphere at 37 °C.

Spheroids of the fibroblasts were obtained using Corning^®^ Ultra Low Adhesion 96-well Black/Clear Round Bottom Microplates (CLS4515-5EA) (Corning, Corning, NY, USA). A total of 600 SC-1 cells were added to each well and cultured for seven days under the same conditions as monolayer in XL-3 CO_2_ incubator (PeCon GmbH, Erbach, Germany). The incubator was connected to a ZEISS Axiovert 200 m microscope (Carl Zeiss AG, Oberkochen, Germany) equipped with an AxioCam MRm camera (Carl Zeiss AG, Oberkochen, Germany). The devices allowed us to photograph the spheroids and measure their size using the AxioVision V4.8.2.0 software program. We used four-day-old spheroids for experiments with AuNPs and MLNCs.

### 2.3. The Preparation of Cell and Spheroid Samples in the Dynamics of Incubation with Nanoparticles

#### 2.3.1. Monolayer

SC-1 cells were seeded in 40 mm diameter Petri dishes (TPP Techno Plastic Products AG, Trasadingen, Switzerland) at a density of 10^5^ cells per dish. Once the monolayer had reached 70% coverage, cells were washed with DMEM, after which AuNPs or MLNCs suspended in 1 mL of DMEM were added. We calculated the concentration of NPs in the culture medium so that it corresponded to 1nM/mL for gold. Serum-free culture medium was used in all experiments with the NPs.

The cells were treated with AuNP or MLNC suspensions in DMEM and incubated for 30 min, 1, 2 or 4 h. After three washes with PBS (Sigma-Aldrich, St. Louis, MO, USA), the cells were removed with trypsin and pelleted by centrifugation for 5 min at 3000 rpm. The resulting pellets were fixed with 4% paraformaldehyde for TEM studies.

#### 2.3.2. Spheroids

Four-day-old SC-1 cell spheroids, which were grown in 96-well plates, were washed with DMEM. AuNPs or MLNCs were mixed with 0.5 mL of DMEM and added to the spheroids. The concentration of NPs in the culture medium corresponded to 1nM/mL for gold. The spheroids were incubated with AuNPs or MLNCs for 1, 2, or 4 h, and fixed with 4% paraformaldehyde. A total of 7–8 spheroids were fixed at each time point of the experiment.

### 2.4. Processing of the Samples for TEM Studies

Transmission electron microscopy reagents were purchased from EMS (Hatfield, PA, USA). Monolayer cells and spheroids were fixed at 4 °C and washed three times with Hanks’ solution (pH 7.4) to remove fixative, then postfixed with 1% osmium tetroxide for one hour. Following a further three washes with Hanks’ solution, the samples were dehydrated in ethanol and acetone in the standard manner, and then embedded in a mixture of Epon and Araldite. The resulting solid blocks were sectioned into ultrathin and semi-thin sections using a diamond knife (Diatome, Nidau, Switzerland) on an EM UC7 ultramicrotome (Leica, Wetzlar, Germany). Semi-thin sections of the spheroids were stained with Azur II, and areas for sharpening pyramids and obtaining ultrathin sections were selected using a Leica DM 2500 light microscope. After contrasting with 2% aqueous solutions of uranyl acetate and lead citrate, the ultrathin sections were examined using a JEM 1400 TEM (JEOL, Tokyo, Japan). Digital images were collected using a Veleta side camera (EM SIS, Münster, Germany).

### 2.5. Scanning Electron Microscopy

Four-day-old SC-1 cell spheroids were fixed in 4% paraformaldehyde (4 °C, 24 h) and after washing with PBS (Sigma-Aldrich, St. Louis, MO, USA), were dehydrated in an ethanol (50%, 70%, 80%, 90%, 96%, and 100%). Then spheroids were immersed in a 1:1 mixture of ethanol and hexamethyldisilazane (HMDS; Sigma-Aldrich, St. Louis, MO, USA) for 10 min, followed by a further 10 min in 100% HMDS. The samples were mounted on a special sample stand with double-sided carbon tape and left to dry for 12–14 h. The spheroids were covered by a 10-nanometer-thick gold/palladium layer using a sputtering device, and then examined in an EVO 10 scanning electron microscope (Carl Zeiss AG, Oberkochen, Germany) at an accelerating voltage of 10 kV.

### 2.6. Measurements

Two solid blocks were obtained from each sample and two grids containing ultrathin sections were prepared from each block. At least 150 cells were examined on each grid and all NP endocytosis events were photographed at the appropriate magnification. In total, at least 200 primary images were obtained for each time point of the experiment.

Semi-thin sections were prepared from all spheroids at each time point at different depths. To obtain ultrathin sections, the pyramids were sharpened at different depths from the outer edge of the spheroid. Two or three grids with ultrathin sections were prepared from each pyramid. All sections (at least 15–20) on each grid were examined, with photographs taken of the spheroid cells and all observed NPs. In total, 200–250 primary images were obtained for each time point.

**Figure 2:**
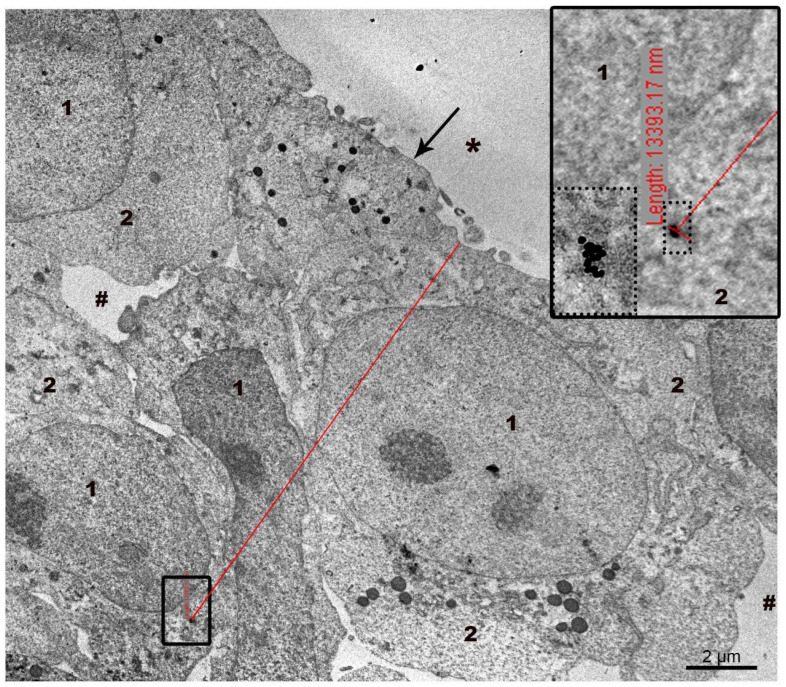
Representative image showing direct measurement of the distance from the detected NP to the edge of the spheroid. NPs found in section is shown by frame, and at higher magnification—by dotted frame. The upper edge of the red line is set at the edge of the spheroid, the lower one—at the detected NP. The line captures two points of interest, and the distance between them is displayed on the screen (a strip of small red marks in a small black frame). In the right corner an image of the inscription at a higher magnification is shown. 1—cell nucleus, 2—cytoplasm, arrow shows plasma membrane, asterisks—outer space, #—intercellular space.

The penetration depth of AuNPs and MLNCs was measured directly on ultrathin sections of spheroids (Figure 2). The viewing of ultra-thin sections of the spheroid was performed radially, from the surface into the depth of the spheroid, in the low-contrast mode of the microscope, which allows one to see electron-dense particles. When the NP was noticed, the viewing mode was changed to standard and the NP was examined and photographed to ensure correct identification. Then the microscope magnification was adjusted to allow the edge of the spheroid and the found NP to be seen in one frame, and a picture was taken. The measurement was carried out on this picture displayed on a screen, using the measuring tool of a digital camera: a point was placed on the edge of the spheroid and a line was drawn with the mouse to the NP, where it was fixed. Next to the second point on the camera screen, the following inscription appeared: “Length” and its value. The penetration depth of other nanoparticles was measured in the same way. The highest values of the penetration depth of the AuNPs and MLNCs were selected from the array of measurements (64 cases). The highest values of the penetration depth of the AuNPs and MLNCs were selected from the array of measurements; the maximum penetration depth of the NPs was 40 µm.

## 3. Results and Discussions

### 3.1. Ultrastructure of SC-1 Fibroblasts in Monolayer

**Figure 3:**
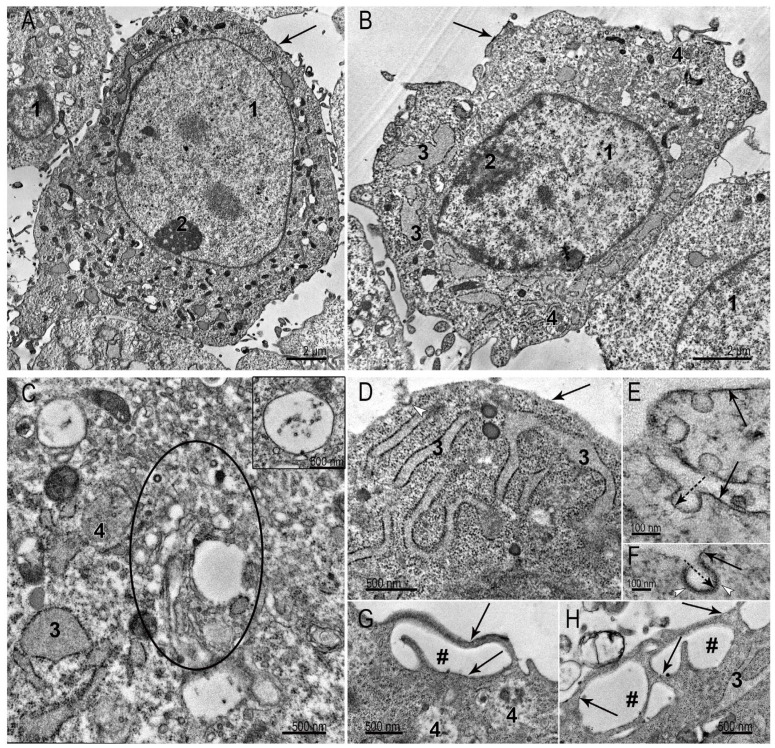
Representative images of SC-1 cells grown in a monolayer. (**A**,**B**) The general appearance of fibroblasts; (**C**) Golgi region (enclosed by oval), the inset presents early endosome, containing few intraluminal vesicles; (**D**) cell area containing cisternae of the ER with medium electron density material; (**E**) plasma membrane invaginations bearing caveolae; dotted arrow shows inner side of caveola membrane; (**F**) clathrin-coated pit; dotted arrow shows inner side of pit membrane; white arrowheads show clathrin “grains” on outer surface of a pit. (**G**,**H**) Cell surface areas showing the macropinocytosis events. 1—nucleus; 2—nucleolus; 3—endoplasmic reticulum; 4—late endosome; #—macropinosome. The arrows point to the plasma membrane.

The SC-1 fibroblasts grown as a monolayer had a spindle-like, sometimes oval shape. TEM examination of the collected monolayer cells reveals a large nucleus with a prominent nucleolus (Figure 3A,B). A distinctive feature of fibroblasts is well-developed protein-synthesizing system, including numerous ribosomes and cisternae of the rough endoplasmic reticulum (ER): intricated network of cisternae of varying length and shape filled with homogeneous material of medium electron density are seen in cytoplasm (Figure 3A–D). Despite the signs of active protein synthesis in SC-1 fibroblasts, signs of extracellular matrix were not detected. The cytoplasm contained all organelles characteristic of eukaryotic cells: mitochondria, Golgi apparatus, intermediate filaments, and all types of endosomes and lysosomes (Figure 3A–D). The Golgi region was rich with coated and smooth vesicles, evidencing high metabolic activity. We carefully searched for signs of clathrin-mediated endocytosis in ultrathin sections of SC-1 fibroblasts in the monolayer, but could detect very rare coated pits on plasma membrane (0–1 in ultrathin section). In contrast, many patterns of macropinocytosis were detected (Figure 3G,H). No signs of intercellular junctions were found.

The plasma membrane holds many caveolae (Figure 3E). Figure 3F shows a clathrin-coated pit, bearing small electron dense “grains” of clathrin on outer membrane surface. It should be noted that the clarity of the “grains” depends on the plane of the cut; if it is not strictly perpendicular, we see an electron-dense strip instead of “grains”. The presence of a large number of caveolae is characteristic of cells that undergo frequent stretching and compression. The endothelial cells, adipocytes, muscle cells, and fibroblasts serve as examples. Caveolae are considered a “reserve” of the plasma membrane, safeguarding cells against mechanical damage during stretching and compression [24].

In general, the ultrastructure of SC-1 fibroblasts in the monolayer demonstrates signs of active metabolism, including protein synthesis and macropinocytosis processes, which seems a main pathway for internalization of external components. The ultrastructure of SC-1 fibroblasts is similar to those described in many studies of culture fibroblasts [25,26].

### 3.2. Ultrastructure of SC-1 Fibroblasts in Spheroids

Our previous study of spheroids composed by HepG2 and HEK293 cells revealed the maintenance of structural polarization of the epithelium, inherent to the “maternal” organ (liver and kidneys, correspondingly). The outer surface of those spheroids was formed only by the basolateral plasma membrane, while the apical zones of the cells were localized inside the spheroids. Spheroids of both types were formed by individual blocks of cells, arranged around bile “capillaries” formed by the apical zones of HepG2 cells, and “clews” of outgrowths of the apical cytoplasm of HEK293 [23]. Fibroblasts do not have the structural polarization characteristic of epithelial tissues, so their spheroids may be organized differently than spheroids of epithelial cells.

#### 3.2.1. Spheroid’s Superficial Layer

Our study focused on the ultrastructural features of fibroblasts, which primarily “meet” NPs introduced into the spheroid in vitro, so some descriptions of cellular organoids may not be detailed enough.

Suspension of fibroblasts, placed in a test tube/Petri dish with a low-adhesive surface, accumulate in a “pile”, from which a spheroid develops. The quantity of seeded cells determines the size of a “freshly formed” spheroid. The spheroid increases in size as the cells that comprise it divide. In this study we used fibroblast spheroids seeded in a dose of 600 cells per well after 4 days of growth, when their diameter reached 350–400 μm. We agreed to consider the superficial layer to be 15–20 μm thick.

**Figure 4:**
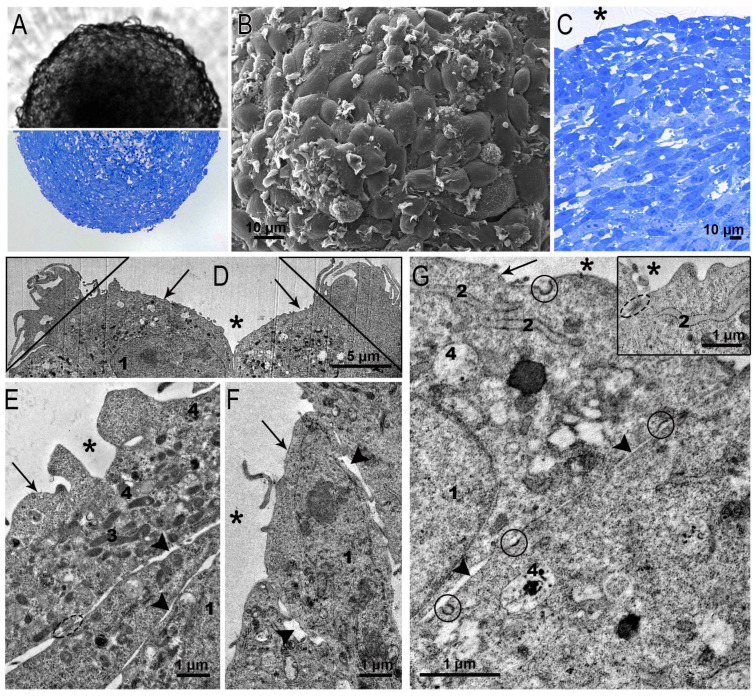
Representative images of squamous SC-1 cells of spheroids. (**A**) SC-1 spheroid in a well (upper part) and on a semi-thin section (bottom part); (**B**) SEM image shows the surface of SC-1 spheroid with a shingles-like organization of cells; (**C**) semi-thin section of the upper area of the SC-1 spheroid showing layered structure; (**D**–**G**) ultrastructure of fibroblasts bordering the external space; the insert in (**G**) shows intercellular junction and ER cisterna. 1—nucleus; 2—endoplasmic reticulum; 3—mitochondria; 4—lysosome; *—external space, arrows indicate plasma membrane; arrowheads—intercellular space, coated pits are enclosed by a circle, dotted circle shows intercellular contact; triangle—folds of micropinocytosis.

SC-1 cell spheroids were perfectly spherical in both the wells and the semi-thin sections (Figure 4A). In SEM, the surface of the SC-1 spheroids looked like fish scales or tiles laid on a roof: the edges of one cell overlapped another. Many surface cells bore flat folds characteristic of the process of macropinocytosis (Figure 4B). The layered arrangement of fibroblasts is clearly visible in semi-thin sections of spheroids (Figure 4C). The layered structure, judging by the published data, is typical for fibroblast spheroids [26,27,28].

Examination of ultrathin sections of squamous fibroblasts bounding spheroids confirmed overlapping of cells (Figure 4D–F) and revealed an interesting feature: the cells showed signs of binding with each other by a kind of adhesive intercellular junctions (Figure 4G, insert). These junctions did not fully correspond to those described in epithelial tissues [29], but undoubtedly contributed to the maintenance of the spherical structure of the spheroids. Junctions were localized between cells at the boundary with the external environment; however, their structure did not allow us to identify them as “typical” occluding junctions. Many small indistinct junctions were seen between the surfaces of adjacent cells (Figure 4E–G). The transition of fibroblasts from the monolayer to the 3D cultivation also led to a change in the plasma membrane: the amount of caveolae significantly reduced. Apparently, this is due to the tension of the fibroblast plasma membrane during the growth of spheroids. The formation of intercellular junctions in spheroids formed by fibroblasts of different origins has also been noted in other studies [25,26,30].

Clear patterns of macropinocytosis were observed on the surface of the squamous surface cells of the SC-1 spheroids in ultrathin sections (Figure 4D), as in the SEM images (Figure 4B). We easily detected coated pits on the plasma membrane of fibroblasts (Figure 4G), indicating clathrin-mediated endocytosis, signs of which were hardly found in the monolayer. The ultrastructure of the nuclei of the squamous fibroblasts in the spheroid did not differ from that observed in the cells of the monolayer. The cytoplasm contained ribosomes, cisterns of the ER, the Golgi apparatus, all types of endosomes, and rare lysosomes (Figure 4E–G).

#### 3.2.2. Ultrastructure of SC-1 Cells in Spheroid’s “Body”

**Figure 5:**
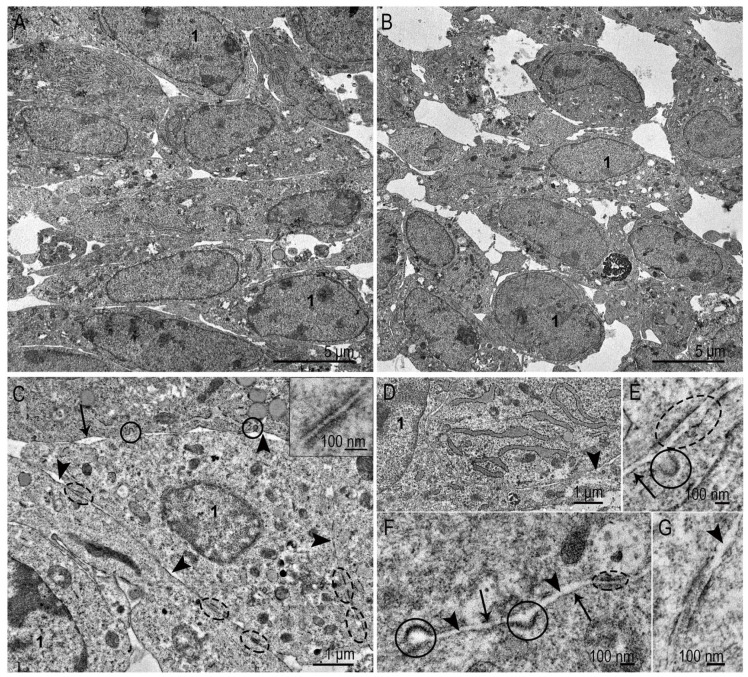
Representative images of spheroid “body”, SC-1 cells. (**A**) Spheroid layers at depth of 25–30 and (**B**) 45–50 microns from the outer surface; (**C**) general view of a fibroblast of spheroid “body”, the insert shows gap junction; (**D**) well-developed endoplasmic reticulum filled with material of middle electron density; (**E**–**G**) gap junctions at different stages of maturation (are enclosed by dotted oval); note fine material of middle electron density between the cells. 1—nucleus, arrows indicate plasma membrane; arrowheads—intercellular space, coated pits are enclosed in circles.

Fibroblasts in the “body” of the spheroid near the surface layer were located densely (Figure 5A), while the deeper “tissue” of the spheroid was loosened (Figure 5B). In the central part (necrosis zone), gradual cell death occurred. Interestingly, the cells were mainly destroyed by apoptosis, and apoptotic bodies were phagocytosed by neighboring fibroblasts. The generally accepted term “necrosis zone” does not correspond to the observed picture in this case. In the “body” of the spheroid, the cells showed no signs of destructive changes. Mitotic figures were evenly distributed both in the surface layer and throughout the entire “body” of the spheroid.

The shape of fibroblasts in the “body” (up to 45–50 μm from the surface) of the SC-1 spheroid was predominantly oval (Figure 5A,B). The ultrastructure of the nuclei and cytoplasm indicated active protein synthesis: numerous ER cisternae filled with homogeneous material of middle electron density were observed; all the previously listed structures were present in the cytoplasm of the spheroid’s “body” (Figure 5A,C). Extracellular matrix components were not detected in the intercellular space. This may be due to the “age” of the spheroids (4 days), since some studies report the appearance of collagen and other elements of the extracellular matrix after 7 or more days of cultivation [26,31,32].

Considering the objective of this work, we specifically investigated the structures potentially involved in the penetration of nanoparticles into the spheroid. The plasma membrane of the fibroblasts in spheroid body was smooth, coated pits (Figure 5C,E,F) were often detected in sections. Caveolae were extremely rare. Numerous gap junctions at various maturation stages (Figure 5C,E–G) connected the cells to each other were observed. In sections of most junctions, we observed deposition of “teeth” of electron-dense substance on the inner side of the plasma membrane and in the intercellular space; we also observed fine fibers and small clumps of medium electron density (Figure 5E,G). The ultrastructure of the junctions looked more “mature” than in the surface layer of the spheroids.

At present, cell spheroids, including fibroblast spheroids, are widely used in various studies. Examples of research can be mentioned in areas such as studies on the effect of agents functionalizing nanoparticles on spheroids [33], changes in the physicochemical characteristics of NPs compared to a monolayer [34], features of fibroblast cell transfection [3], and toxicity of a new preparations [2]. Changes in the structure of fibroblasts during the transition from a monolayer to a three-dimensional form include such significant changes as a decrease in the number of caveolae and a “straightening” of the plasma membrane, which undoubtedly leads to a reorganization of the molecular structure of the plasma membrane, due to the exposure of caveolae membrane molecules to the surface. Another reorganization is the emergence and “maturation” of intercellular contacts, which can prevent the migration of AuNPs and M through the intercellular spaces. This point will be studied further.

### 3.3. Interaction of AuNPs and MLNCs with SC-1 Cells in a Monolayer

Before moving on to the results of our NP research, it is necessary to convey to the reader a number of features of studying ultrathin sections in a transmission electron microscope. Specifically, the “working” thickness of ultrathin sections is 70–80 nm (1 nm equals one billionth of a meter (10^−9^ m). The cell sizes vary; we will assume that they are on average 10 µm (1 µm equals 10^−6^ m). The sizes of the NPs vary; the NPs in this study have a diameter of 12–14 nm. The researcher must study both the cells and the NPs in one section. The reliability of the results is achieved by viewing a large number of ultra-thin sections passing through each cell. Obviously, there is a question of correct identification of the cellular structures, and this is not always possible. Another complicating factor is the plane of the section: if it is not perpendicular to the structure, the membranes “disappear” due to the physical effects of the passage of electrons through the section. However, only TEM of ultrathin sections allows one to simultaneously see both NPs and cellular structures and evaluate their interaction, which makes it an indispensable tool in nanobiology.

**Figure 6:**
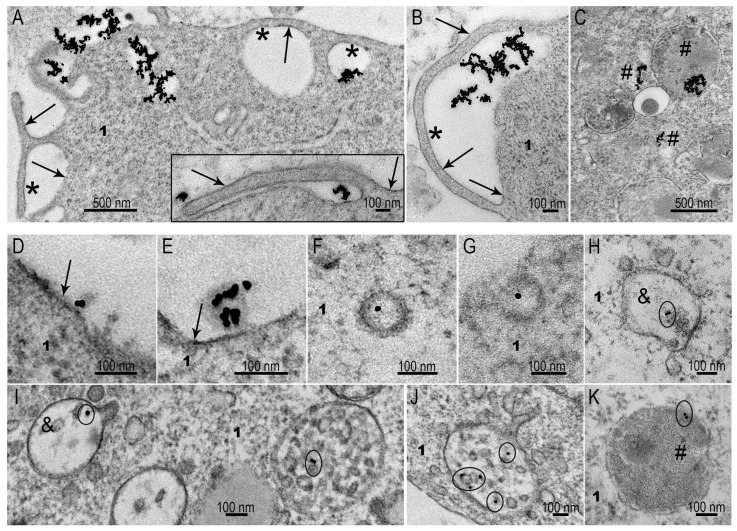
Representative images of SC-1 monolayer cell interaction with AuNPs and MLNCs. (**A**,**B**) Macropinocytosis of AuNP aggregates of different sizes by SC-1 fibroblasts; (**C**) AuNP aggregates internalized by micropinocytosis; (**D**,**E**) adsorption of MLNCs on plasma membrane of SC-1 fibroblasts; (**F**,**G**) coated vesicles containing a MLNC; (**H**) early endosome, containing a MLNC; (**I**–**K**) early and late endosomes containing MLNCs. 1—cytoplasm; arrows indicate plasma membrane; *—cavity of macropinosome; #—lysosomes; &—early endosome. The circles in early and late endosomes show visible AuNPs inside these structures.

SC-1 fibroblasts showed active uptake of AuNPs by macropinocytosis, and plasma membrane folds internalized accumulations of AuNPs, forming macropinosomes (Figure 6A–C). Coated pits containing AuNPs were exceptional finding on the plasma membrane of SC-1 fibroblasts in the monolayer, as well as early endosomes containing AuNPs (not more than 1–2 cases in 100 cells). Very rarely, AuNPs were found at the “entrance” to caveolae, not inside. We interpret the presence of NPs at the caveolae “entrance” as accidental adsorption of the particles. Thus, macropinocytosis seems to be a main way of citrate AuNP penetration into SC-1 fibroblasts in the monolayer.

The ability of SC-1 fibroblasts to clathrin-mediated endocytosis was fully demonstrated during the interaction of cells with MLNCs (Figure 6D–K). Coated pits (Figure 6F,G) and early endosomes (Figure 6H,I) were frequently observed in ultrathin sections (3–10 cases in 50 cells). These structures represent initial steps of clathrin-mediated endocytosis [18,19,35], and their presence evidence for “complete” process of the enocytosis. A feature of clathrin-mediated endocytosis of MLNCs was absence of particle aggregation, such that they remained individual units at all steps of endocytosis, including late endosomes and lysosomes (Figure 6I–K).

The obtained results showed the ability of monolayer fibroblasts to selectively adsorb different types of NPs. It should be noted that these phenomena were observed in the absence of serum, when there was no formation of a protein corona.

The ability of SC-1 fibroblasts to clathrin-mediated endocytosis was fully demonstrated during the interaction of cells with MLNCs (Figure 6D–K). Coated pits (Figure 6F,G) and early endosomes (Figure 6H,I) were frequently observed in ultrathin sections. These structures represent initial steps of clathrin-mediated endocytosis [18,19,35]. A feature of clathrin-mediated endocytosis of MLNCs was absence of particle aggregation, whereby they retained their individuality at all steps of endocytosis, including late endosomes and lysosomes (Figure 6I–K).

The obtained results showed the ability of monolayer SC-1 fibroblasts to selectively uptake different types of NPs. It should be noted that these phenomena were observed in the absence of serum, when there was no formation of a protein corona.

### 3.4. Interaction of AuNPs with Spheroid SC-1 Cells

**Figure 7:**
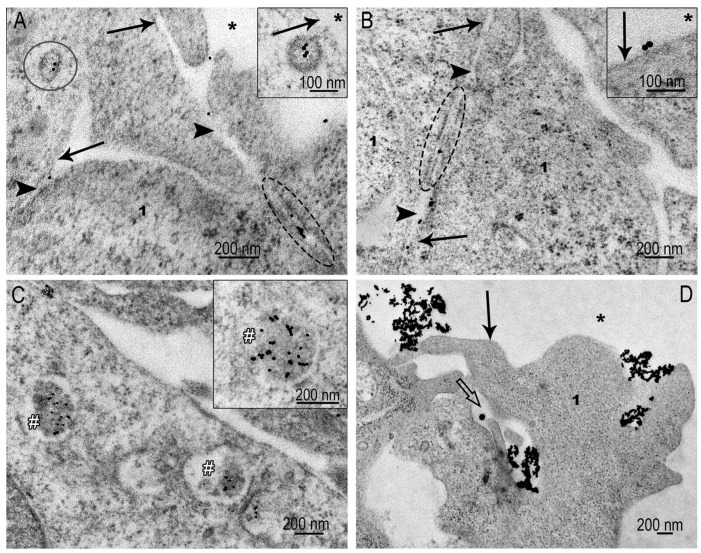
Representative images of AuNP interaction with SC-1 cells of spheroid. (**A**,**B**) Adsorption of individual AuNPs on plasma membrane and coated pits; migration of AuNPs through gap junction; note: insets show coated pit containing AuNPs and adsorption of AuNPs on plasma membrane. (**C**) Accumulation of individual AuNPs in lysosomes indicated with #; (**D**) phagocytosis of AuNP clusters by spheroid cells, and white arrows show cell protrusions forming phagosome. Plasma membrane is indicated by arrow; coated pit is indicated by circle; asterisk shows outer space; dotted oval indicates area of gap junctions; 1—cytoplasm.

Clathrin-mediated endocytosis seems the dominant pathway for AuNP uptake by spheroid cells. Coated pits were easily detected on the outer and lateral surfaces of the fibroblasts (Figure 7A–C), indicating their significant number. AuNPs were individual units, or in groups of 2–3 particles. Interestingly, the AuNPs migrated through intercellular junctions, which apparently did not pose significant obstacles to their movement (Figure 7A,B).

Late endosomes and lysosomes containing AuNPs were found in many spheroid cells after 4 h of incubation, indicating the accumulation of AuNPs in cells and a fairly high intensity of clathrin-dependent endocytosis (Figure 7C).

Macropinocytosis events, which were frequent in monolayer cells (Figure 6), were not detected in spheroid sections. We propose that the plasma membrane stretched on the spheroid surface is unable to form appropriate folds to capture AuNPs. AuNPs of suitable size for clathrin-mediated endocytosis use this pathway. However, we observed rare patterns that can be identified as phagocytosis (Figure 7D) of AuNP aggregates.

### 3.5. Interaction of MLNCs with Spheroid SC-1 Cells

The MLNCs we created are unique nanocomposites carrying siRNA and coated with a lipid envelope. We demonstrated the efficiency of siRNA delivery via MLNCs using confocal microscopy—siRNA blocked the expression of GFP in HEK Phoenix cells with stable integration of GFP gene into the genome [7].

**Figure 8:**
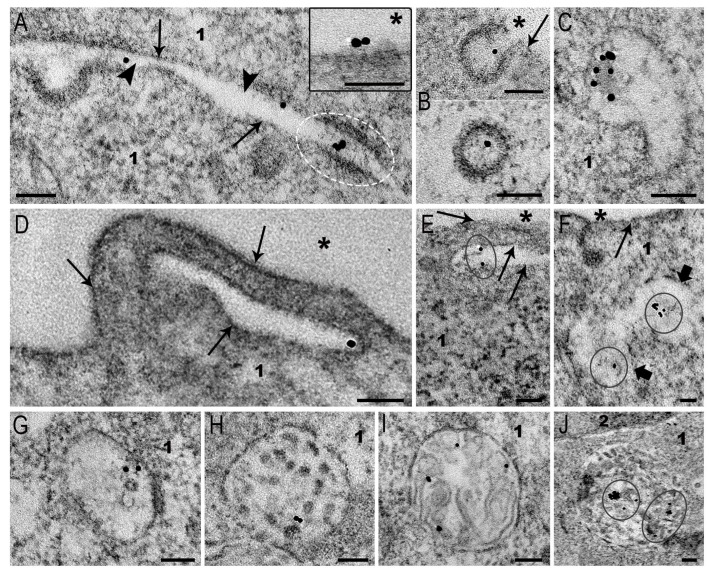
Representative images of MLNC endocytosis events in SC-1 fibroblast spheroids. (**A**) Adsorption of MLNCs on plasma membrane; MLNCs in the space between two cells; the particles inside intercellular junction are shown by white dotted oval. The inset shows adsorption of AuNPs on a surface plasmalemma; (**B**) longitudinal and cross sections of the coated pits; (**C**) early endosome; (**D**–**F**) macropinocytosis of MLNCs; (**G**–**J**) accumulation of MLNCs in endosomes and lysosomes. 1—cytoplasm; 2—nucleus, plasma membrane is shown by arrows, intercellular space is shown by arrowheads, external space is shown by asterisks, macropinosome membrane is indicated with thick arrows, and AuNPs at the low magnification are enclosed in circles. Length of scale bars corresponds to 100 nm.

Spheroid fibroblasts took up MLNC by clathrin-mediated endocytosis, similarly to monolayer fibroblasts. The patterns of endocytosis were observed at all time points, being more frequent at 4 h of incubation. Figure 8A,B demonstrate MLNC adsorption and their localization in coated pits. MLNCs, similarly to AuNPs, migrated through intercellular gap junctions between spheroid cells (Figure 8A). The early endosome is the first “station” where endocytic vesicles carrying internalized particles arrive, and a place of many cargos and viral RNA release [18,19,35]. These structures containing MLNCs can be seen in Figure 8C,G.

Signs of MLNC macropinocytosis in spheroid fibroblasts (Figure 8D–F) were exceptionally rare (all found events are presented in the image). The rarity of detection does not allow us to analyze features of MLNCs macropinocytosis in spheroids. It is possible to conclude that this route of endocytosis does not play an appreciable role in MLNC internalization by spheroid cells.

As is known, all endocytic pathways end in late endosomes and lysosomes, where the endocytosed material is cleaved by hydrolases [17,18,19]. We observed the accumulation of MLNCs in early and late endosomes (Figure 8C,G–I) of fibroblast spheroids. In lysosomes, MLNCs showed tendency to fusion, forming small clusters (Figure 8J). This probably reflects a violation of the integrity of the MLNCs’ lipid envelope.

The obtained results show that MLNCs enter fibroblast spheroids via clathrin-dependent endocytosis. We determined the depth to which MLNCs penetrated the spheroids in 4 h: the depth was 35–40 μm, as in the case of AuNPs. We believe that penetration into the spheroid is mainly provided by NP migration through intercellular spaces. The ability of AuNPs and MLNCs to “pass” through intercellular junctions represent an interesting phenomenon that has not been described before and, undoubtedly, needs further investigation. Indeed, we clearly see MLNC inside the filaments that connect the cells (Figure 8A), and it is not clear what mechanisms provide this.

We studied the penetration of AuNPs and MLNCs into spheroids and found that AuNPs and MLNCs migrated through the intercellular spaces; however, their movement was stopped at a level of about 40 µm from the surface, while the diameter of spheroids was 350–400 µm. We previously examined the penetration of AuNPs, BSA-AuNPs, and PEI-AuNPs into HEK293 and HepG2 spheroids, and detected the same value of NP immersion into spheroids: about 40 µm from the surface [23]. It should be noted that HEK293 cells are not cancerous, just like fibroblasts. According to published data, the penetration depth of various nanoparticles into different spheroids varies from 30 to 70 µm. The factors influencing the movement of NPs include the size, charge, and shape of the NPs, the density of the intercellular matrix, and the increase in interstitial pressure inside the spheroid [36,37,38,39,40]. In the spheroids of fibroblasts and other cells that we studied, there is no extracellular matrix, and the particles are similar in properties. This gives us grounds to believe that the force that stops the migration of AuNPs, MLNCs, BSA-AuNPs, and PEI-AuNPs is an increase in interstitial pressure.

Clathrin-mediated endocytosis of AuNPs, and their modifications, has been demonstrated in many studies [19,20,33]. However, the selectivity of fibroblasts towards AuNPs had not been reported in the literature, and should be taken into account when planning experiments using these NPs. In this paper, we provide direct evidence of active clathrin-mediated endocytosis of MLNCs by SC-1 fibroblasts in spheroids and a monolayer in serum-free culture medium, and these results are positive for our further studies.

## 4. Conclusions

Both MLNCs and AuNPs penetrated into spheroids to a depth of 35–40 μm through the intercellular spaces. We have shown for the first time the free movement of AuNPs and MLNCs through intercellular junctions in fibroblast spheroids.

The internalization pathways of AuNPs differed depending on the fibroblast culture form: in the monolayer, macropinocytosis was mainly detected, whereas in the spheroid form, it was clathrin-dependent endocytosis.

We observed direct evidence of MLNCs clathrin-mediated endocytosis in SC-1 fibroblasts: adsorption, formation of coated pits and presence of the particles in early and late endosomes, and accumulation in lysosomes. These events did not differ in monolayer cells and spheroids, and were detected equally during 4 h of incubation.

The experiments were conducted in serum-free medium, so the influence of the protein corona was excluded.

## Data Availability

The data is available upon contacting the corresponding author.
